# Anti-Inflammatory and Antioxidant Effects Induced by *Allium sativum* L. Extracts on an Ex Vivo Experimental Model of Ulcerative Colitis

**DOI:** 10.3390/foods11223559

**Published:** 2022-11-09

**Authors:** Lucia Recinella, Era Gorica, Annalisa Chiavaroli, Caterina Fraschetti, Antonello Filippi, Stefania Cesa, Francesco Cairone, Alma Martelli, Vincenzo Calderone, Serena Veschi, Paola Lanuti, Alessandro Cama, Giustino Orlando, Claudio Ferrante, Luigi Menghini, Simonetta Cristina Di Simone, Alessandra Acquaviva, Maria Loreta Libero, Nilofar, Luigi Brunetti, Sheila Leone

**Affiliations:** 1 Department of Pharmacy, G. d’Annunzio University of Chieti-Pescara, 66013 Chieti, Italy; 2 Department of Pharmacy, University of Pisa, 56126 Pisa, Italy; 3 Department of Drug Chemistry and Technology, Sapienza University of Rome, 00185 Rome, Italy; 4 Interdepartmental Research Center “Nutrafood: Nutraceutica e Alimentazione per la Salute”, University of Pisa, 56126 Pisa, Italy; 5 Interdepartmental Research Center “Biology and Pathology of Ageing”, University of Pisa, 56126 Pisa, Italy; 6 Department of Medicine and Aging Science, Centre on Aging Sciences and Translational Medicine (Ce.S.I-MeT), University “G. d’Annunzio” of Chieti-Pescara, 66100 Chieti, Italy; 7 Veridia Italia Srl, Via Raiale 285, 65100 Pescara, Italy

**Keywords:** garlic, colon, multimethodological evaluation, CIEL*a*b*, HS–SPME/GC–MS, apoptosis, inflammatory bowel disease

## Abstract

Inflammatory bowel diseases (IBDs) are chronic and multifactorial inflammatory conditions of the colonic mucosa (ulcerative colitis), characterized by increased and unbalanced immune response to external stimuli. Garlic and its bioactive constituents were reported to exert various biological effects, including anti-inflammatory, antioxidant and immunomodulatory activities. We aimed to evaluate the protective effects of a hydroalcoholic (GHE) and a water (GWE) extract from a Sicilian variety of garlic, known as Nubia red garlic, on an ex vivo experimental model of ulcerative colitis, involving isolated LPS-treated mouse colon specimens. Both extracts were able to counteract LPS-induced cyclooxygenase (COX)-2, tumor necrosis factor (TNF)-α, nuclear factor-kB (NF-kB), and interleukin (IL)-6 gene expression in mouse colon. Moreover, the same extracts inhibited prostaglandin (PG)E_2_, 8-iso-PGF_2α_, and increased the 5-hydroxyindoleacetic acid/serotonin ratio following treatment with LPS. In particular, GHE showed a better anti-inflammatory profile. The anti-inflammatory and antioxidant effects induced by both extracts could be related, at least partially, to their polyphenolic composition, with particular regards to catechin. Concluding, our results showed that GHE and GWE exhibited protective effects in colon, thus suggesting their potential use in the prevention and management of ulcerative colitis.

## 1. Introduction

Garlic (*Allium sativum* L.) is an herbaceous plant, belonging to the Amarillidaceae family, that is used all over the world as traditional medicine and spice [1]. Garlic contains a number of biologically active compounds, including phenolic compounds [2], saponins [3], polysaccharides [4], as well as organosulfur compounds [1] that contribute to its countless pharmacological properties. In particular, the health-promoting properties induced by garlic were suggested to be related to its bioactive compounds, including phenolic compounds, whose content in this plant is relatively high [5]. However, the content of phenolic compounds in garlic was reported to be dependent on environmental, genetic and agronomic factors [6]. Preclinical and clinical studies demonstrated the anti-inflammatory, antioxidant, antiatherosclerotic, anticancer, immunomodulatory, antidiabetic, antiobesity, neuroprotective, as well as digestive system protective activities of both garlic and its phenolic constituents [1]. Interestingly, raw garlic showed higher antioxidant activity compared to cooked garlic [7], suggesting that the antioxidant property of garlic could be modified by the processing methods. Moreover, in vitro and in vivo studies showed that garlic could suppress inflammation mainly through inhibition of various inflammatory biomarkers, including nitric oxide (NO), tumor necrosis factor (TNF)-α, and interleukin (IL)-1 [1].

The beneficial effects induced by garlic were also be related to its organosulfur compounds, including diallyl thiosulfonate (allicin), diallyl sulfide (DAS), diallyl disulfide (DADS), diallyl trisulfide (DATS), E/Z-ajoene, S-allyl-cysteine (SAC), as well as S-allyl-cysteine sulfoxide (alliin) [1]. In particular, allicin was reported to be among the most important bioactive constituents exerting antioxidant effects [8].

Inflammatory bowel diseases (IBDs) are chronic and multifactorial inflammatory conditions of the colonic mucosa (ulcerative colitis), characterized by increased and unbalanced immune response to external stimuli [9,10,11]. In this context, antioxidant/anti-inflammatory herbal extracts were found to contrast IBD-related symptoms [12,13] by reducing various pro-inflammatory and oxidative biomarkers, such as reactive oxygen/nitrogen (ROS/RNS) species, prostaglandins, and cytokines.

Interestingly, garlic was suggested to exert protective effects against ulcerative colitis [14]. In addition, garlic oil suppressed endotoxin-induced neutrophil infiltration in small intestine of rats [15]. Moreover, Balaha and collaborators (2016) [16] showed that garlic oil (GO) was able to inhibit ulcerative colitis induced by dextran sulfate sodium in rats. This effect was suggested to be related to antioxidant, anti-inflammatory as well as to immunomodulatory effects of GO. Accordingly, more recently, GO was reported to decrease inflammation and cellular damage in a rat model of colitis induced by acetic acid [17].

On the basis of these findings, in this study, we aimed to evaluate the potential protective effects of a hydroalcoholic extract (GHE) and a water extract (GWE) of garlic on isolated mouse colon specimens treated ex vivo with *E. coli* lipopolysaccharide (LPS), which represents an experimental model of ulcerative colitis [18]. In particular, our research focused on hydroalcoholic and water extracts from a Sicilian variety of garlic (Nubia red garlic), known for the intense red color of the robes of its bulbils [19]. This variant is a protected denomination of origin product (DOP), which is appreciated all over the world. We reported here in, to the best of our knowledge, the first potential beneficial effects of Nubia red garlic on colon inflammation.

## 2. Materials and Methods

### 2.1. Preparation of Garlic Extracts

Garlic cloves were supplied as dried powder by il Grappolo S.r.l. (Soliera, Modena, Italy). Plant sample (1 g) was mixed with either a solution of ethanol–water (20:80, *v/v*) or water (final concentration = 1 g/mL), as previously reported [20,21,22]. The supernatant was filtered and then dried (freeze-drying). The dry residue, a yellow sugary solid, was stored at 4 °C, until chemical analyses were performed.

### 2.2. High Performance Liquid Chromatography (HPLC)–Diode Array (DAD)–Mass Spectrometry (MS) Analysis

Selected phenolic compounds contained in the extracts were identified and quantified by HPLC–DAD–MS analysis. The HPLC apparatus consisted of a two PU-2080 PLUS chromatographic pump, a DG-2080-54 line degasser, a mix-2080-32 mixer, UV, diode array (DAD) and detectors, a mass spectrometer (MS) detector (expression compact mass spectrometer (CMS), Advion, Ithaca, NY, USA), an AS-2057 PLUS autosampler, and a CO-2060 PLUS column thermostat (all from Jasco, Tokyo, Japan). Integration was conducted through ChromNAV2 Chromatography software. The separation was performed on an Infinity lab Poroshell 120-SB reverse phase column (C18, 150 × 4.6 mm i.d., 2.7 µm) (Agilent, Santa Clara, CA, USA). Column temperature was set at 30 °C. The separation was conducted within 60 min of the chromatographic run, starting from the following separation conditions: 97% water with 0.1% formic acid, 3% methanol with 0.1% formic acid, as previously described [23]. Quantitative determination of phenolic compounds was conducted through a DAD detector. Qualitative analysis of GHE and GWE was performed by an MS detector in the positive and negative ion modes. MS signal identification was performed by comparison with a standard solution and MS spectra available in the MassBank Europe database (https://massbank.eu/MassBank/ (accessed on 11 November 2021). All HPLC grade solvents were purchased from Merck Science Life S.r.l. (Milan, Italy). Each analysis was performed in triplicate. The detailed protocol is enclosed as supplementary materials. 

### 2.3. Headspace Solid-Phase Microextraction-Gas Chromatography–Mass Spectrometry (HS–SPME–GC–MS) Analysis

The 4 mL vials were loaded with 50 mg of the garlic dry residue. According to the extraction parameters optimized in our previous screening study performed on the garlic powder [15], the sample was allowed to equilibrate at 80 °C for 20 min and then the SPME fiber DVB-CAR-PDMS (Sigma Aldrich, now Merck KGaA, Darmstadt, Germany) was exposed to the head space of the vial for 20 min at 80 °C.

After sampling, fiber was withdrawn in the needle and exposed into the GC inlet at 260 °C for 0.5 min. The desorbed analytes were introduced in a gas chromatograph (6850, Agilent Technologies, Santa Clara, CA, USA) coupled with a mass spectrometer (5975, Agilent Technologies, Santa Clara, CA, USA), equipped with the non-polar capillary column HP-5MS (30 m × 0.25 mm inner diameter, and film thickness 0.25 µm). The gas-chromatographic parameters were set as follows: inlet temperature, 260 °C; injection mode, splitless (splitter valve was opened after 0.2 min and split ratio = 20/1); flow rate of the helium carrier gas (99.995% purity), 1.0 mL/min; oven temperature starting from 40 °C, after 5 min raised to 200 °C at 5 °C/min, and kept at this final temperature for 60 min. Mass spectrometry parameters were set as follows: EI energy, 70 eV; source temperature, 230 °C; quadrupole temperature, 150 °C; and mass scan was carried out over the 50–350 *m*/*z* range. The analyses were performed in triplicate.

Two analytical criteria were used to allow the identification of the eluted compounds, namely the comparison between the EI experimental spectra and those collected in the commercial (FFNSC 3) and free access databases (NIST 11, Flavor2) and the Kovats index (KI) measured using a mixture of *n*-alkanes (C7–C40) with the same chromatographic conditions, and then compared with values reported in the FFNSC 3 and NIST 11 databases. A manual integration of chromatographic peaks with a S/N ratio above 3 was performed without any further modification.

### 2.4. Positive-Ion Direct Infusion–Electrospray Ionization–Mass Spectrometry (DI–ESI–MS) Analysis

The dry residue of garlic was dissolved in H_2_O:MeOH (7:3) to a final concentration of 20 μg/mL and directly infused at 10 μL min^−1^ in the ESI source of a LTQ XL linear ion trap (Thermo Fisher Scientific, Waltman, MA, USA). The source parameters were set as follows: source voltage = 4.5 kV; capillary voltage = 15 V; capillary temperature = 300 °C; tube lens voltage 89.9 V, sheath gas flow rate 10 (arbitrary units). Each spectrum, acquired over the 170–2000 *m/z* range, was from averaging 10 full scans, each one consisting of 5 micro scans. The major peaks were isolated and submitted to MS tandem experiments to allow the compound identification by comparing their relevant MS/MS spectra with those reported in literature or collected in a free access database (https://massbank.eu/MassBank/Search (accessed on 19 May 2022). The precursor ion isolation width was 1–2 Da and the normalized collision energy was set to the value needed to reduce the intensity of the precursor ion to approximately 10%.

### 2.5. Colorimetric Analysis

Colorimetric analysis of garlic powder (GP) sample and GWE was performed by X-Rite MetaVueTM^®^ (Prato, Italy) as previously described [15]. GP sample and GWE analysis was conducted at the time of delivery (t°) and after 12 months (t^12m^) of storage in the darkness at room temperature (25 ± 2 °C). The detailed description of colorimetric analysis is reported in the Supplementary Materials section.

### 2.6. HPLC–DAD Analysis

GWE was weighed, dissolved in water and filtered before injection into a HPLC Perkin Elmer apparatus (Series 200 LC pump, Series 200 DAD and Series 200 autosampler, Milan, Italy). Chromatography was conducted on RP-18 column (3 µ) using a linear gradient consisting of acetonitrile and acidified water (5% formic acid), from 100% aqueous phase to 85% in 15 min, 85 to 55% in 30′ and 55 to 40% in 20′, at a flow rate of 0.8 mL/min, at 254 nm. Alliin was quantified as previously described [22] (y = 6.35x + 50.34; R2 = 0.9987, in the range between 2 and 400 µg/mL, LOD 0.6 µg/g and LOQ 2.0 µg/g extract in dry weight). Each analysis was performed in quadruplicate. The detailed protocol related to HPLC-DAD analysis is described in the Supplementary Materials section.

### 2.7. Cell Lines

Colorectal cancer cell line SW480 was cultured in RPMI1640 (Sigma, St. Louis, MO, USA) supplemented with 10% fetal bovine serum (FBS), 1% Pen/Strep and 1% L-glutamine, and maintained in a humidified incubator at 37 °C, 5% CO_2_.

### 2.8. Cell Viability Assay

Evaluation of cell viability was performed by MTT assay [3-(4,5-dimethyl-2-thiazolyl)-2,5-diphenyl-2H-tetrazolium bromide] (Sigma, St. Louis, MO, USA) as previously reported [24]. SW480 cell line was seeded in 96-well plates (5 × 10^3^ cells/well) and was treated the following day with GHE (1, 10, and 100 μg/mL), GWE (1, 10, and 100 μg/mL) or vehicle (culture medium). Each analysis was performed in triplicate.

### 2.9. Apoptosis Assay

To evaluate apoptosis, BD Pharmingen™ APC Annexin V antibody (BD, Becton-Dickinson Biosciences, San Jose, CA, USA) and propidium iodide (PI) (Sigma, St. Louis, MO, USA) were used according to the manufacturer’s instructions, essentially as previously described [25]. Each analysis was performed in triplicate

### 2.10. Ex Vivo Studies

Adult C57/BL6 male mice (3-month-old, weight 20–25 g) were housed in Plexiglas cages (2–4 animals per cage; 55 cm × 33 cm × 19 cm) and maintained under standard laboratory conditions. Experimentation procedures were in agreement with the European Community ethical regulations (EU Directive no. 26/2014) on the care of animals for scientific research and approved by local ethical committee (‘G. d’Annunzio’ University, Chieti, Italy) and Italian Health Ministry (Project no. 885/2018-PR).

After collection, isolated colon specimens were maintained in a humidified incubator with 5% CO_2_ at 37 °C for 4 h (incubation period) in RPMI buffer with added bacterial LPS (10 μg/mL) [26,27] and treated with either GHE or GWE (1, 10, and 100 μg/mL). Prostaglandin (PG) E_2_ and 8-iso-PGF_2α_ levels (pg/mg wet tissue) were measured by radioimmunoassay (RIA) in tissue supernatants [28,29]. Each analysis was performed in triplicate.

Extraction of total RNA from colon specimens was performed using TRI reagent (Sigma-Aldrich, St. Louis, MO, USA), according to the manufacturer’s instructions. Reverse transcription was performed using High Capacity cDNA Reverse Transcription Kit (ThermoFischer Scientific, Waltman, MA, USA). Gene expression of cyclooxygenase (COX)-2, TNF-α, nuclear factor-*k*B (NF-*k*B), IL-6, and nuclear factor erythroid 2-related factor 2 (Nrf2) was measured by quantitative real-time PCR using TaqMan probe-based chemistry [30,31]. The real-time PCR was performed in triplicate. Relative quantification of gene expression was conducted through the comparative 2^−ΔΔCt^ method [32]. The detailed description of real-time PCR is reported in the Supplementary Materials section.

Extraction of serotonin (5-HT), and 5-hydroxyindolacetic acid (5HIIA) was performed from individual colon specimens homogenized in perchloric acid solution (50 mM). Analysis of colon 5-HT, and 5HIIA levels was performed through high performance liquid chromatography coupled to electrochemical detection consisting of ESA Coulochem III detector equipped with ESA 5014B analytical cell [33,34]. Each analysis was performed in triplicate.

### 2.11. Statistical Analysis

Analysis of the data was performed by using the software GraphPad Prism version 6.0 (Graphpad Software Inc., San Diego, CA, USA). Means ± SEM were assessed for each experimental group and analyzed by one-way analysis of variance (ANOVA), followed by the Newman–Keuls multiple comparison post hoc test. As for quantification of the investigated phenolic compounds detected in GHE and GWE, analysis of the data was conducted by unpaired *t* test (two-tailed *p* value). The limit of statistically significant differences between mean values was set at *p*-value < 0.05. Calculation of the number of animals randomized for each experimental group was performed by using the “Resource Equation” *n* = (E + T)/T (10 ≤ E ≤ 20) [35].

## 3. Results and Discussion

### 3.1. Phytochemical Analyses

#### 3.1.1. HPLC–DAD–MS Analysis

**Figure 1:**
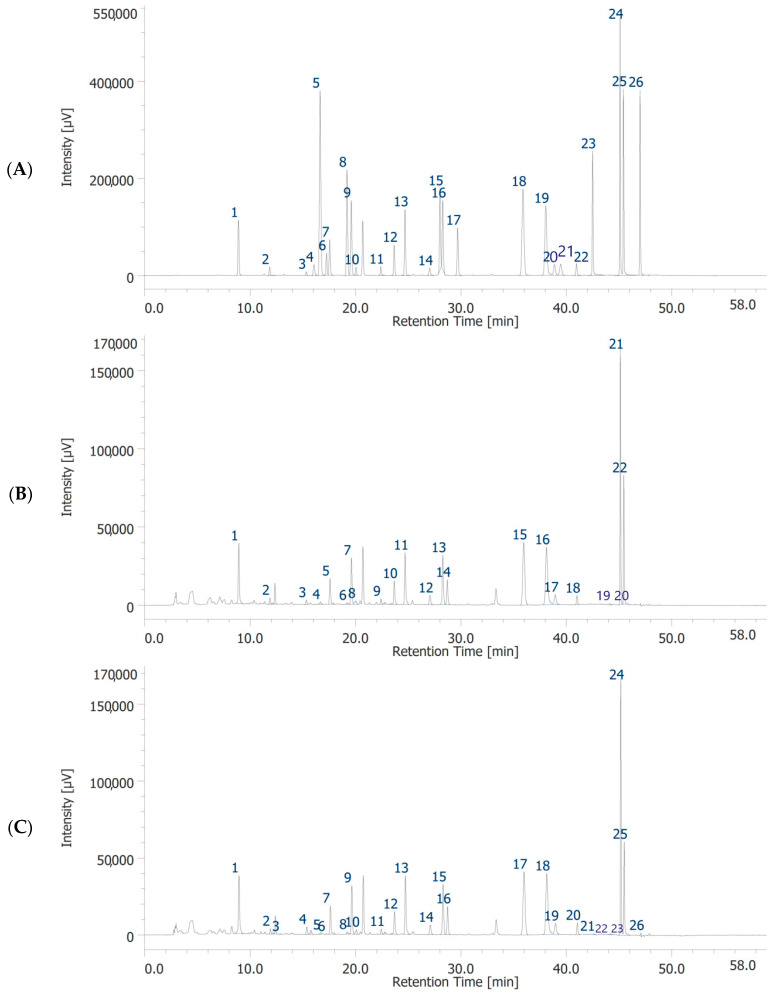
HPLC-DAD chromatograms of standards solution (**A**), garlic water extract (GWE) (**B**) and garlic hydroalcoholic extract (GHE) (**C**). Chromatographic analysis of GWE (**B**). The chromatographic analysis showed the presence of 18 phytochemicals: gallic acid (peak #1), 3-hydroxytyrosol (peak #2), catechin (peak #3), chlorogenic acid (peak #5), caffeic acid (peak #7), epicatechin (peak #8), syringaldehyde (peak #9), p-coumaric acid (peak #10), t-ferulic acid (peak #11), benzoic acid (peak #12), rutin (peak #13), resveratrol (peak #14), t-cinnamic acid (peak #15), quercetin (peak #16), naringenin (peak #17), hesperetin (peak #18), flavone (peak #21), 3-hydroxyflavone (peak #22). Chromatographic analysis of GHE (C). The chromatographic analysis showed the presence of 19 phytochemicals: gallic acid (peak #1), 3-hydroxytyrosol (peak #2), caftaric acid (peak #3), catechin (peak #4), chlorogenic acid (peak #7), caffeic acid (peak #9), epicatechin (peak #10), syringaldehyde (peak #11), p-coumaric acid (peak #12), t-ferulic acid (peak #13), benzoic acid (peak #14), rutin (peak #15), resveratrol (peak #16), t-cinnamic acid (peak #17), quercetin (peak #18), naringenin (peak #19), hesperetin (peak #20), flavone (peak #24), 3-hydroxyflavone (peak #25).

Both garlic extracts were analyzed for the measurement of the levels of selected flavonoids and phenols by quantitative HPLC–DAD–MS analysis. The list of the polyphenolic compounds studied, as well as the retention time, wavelength, and the *m*/*z* ratio for their determination are reported in Table S2. Quantification was performed as previously reported [23]. The characterization of the investigated phenolic compounds is reported in Table 1. A variety of factors influence composition of garlic extracts, such as source of garlic strain, conditions of storage, processing type, and aging [36,37]. The biological activity of garlic components has also been reported to be dependent on extraction, temperature, preparation, and storage [38]. In addition, the data are not easily comparable with literature, being chiefly related to the garlic clove and garlic powder rather than to the separated parts [39,40]. The chromatographic analysis of GHE and GWE confirmed the presence of 19 and 18 phytochemicals, respectively (Figure 1 and Table 1). In the present study, the prominent compound found in GHE was catechin, while the prominent compounds found in GWE were catechin and benzoic acid. Gallic acid, chlorogenic acid, *p*-coumaric acid, *t*-ferulic acid, benzoic acid, resveratrol, naringenin, hesperetin, and flavone were present at concentrations ranging from 22.66 to 13.33 μg/mL in GHE, and from 12.73 to 21.51 μg/mL in GWE. On the other hand, concentrations of 3-hydroxytyrosol, caffeic acid, epicatechin, syringaldehyde, *p*-coumaric acid, *t*-cinnamic acid, quercetin, and 3-hydroxyflavone ranged from 8.94 to 11.83 μg/mL in GHE, and from 7.23 to 11.59 μg/mL in GWE. Caftaric acid was detected only in GHE, at very lower concentration (0.93 μg/mL). Interestingly, GHE was richer than GWE in catechin, epicatechin, *t*-ferulic acid, benzoic acid, resveratrol, quercetin, naringenin, and hesperetin. On the other hand, GWE showed higher levels of 3-hydroxytyrosol and 3-hyroxyflavone as compared to GHE.

#### 3.1.2. HS–SPME–GC–MS and DI–ESI–MS Analysis

The compounds identified through the HS–SPME–GC–MS were clustered in three main classes (Table 2), namely the monoterpenes and their oxygen derivatives (61.2%), the sulfur-containing-compounds (SCC, 22.8%) and finally the aldehydes (3%). Thymol and carvacrol, two monoterpenoids, are the most abundant species (22.4% and 36.1%, respectively), followed by allyl disulfide (9.52%) and the 3-vinyl-1,2-dithiacyclohexene isomers (7.14%) belonging to the SSC class.

**Figure 2:**
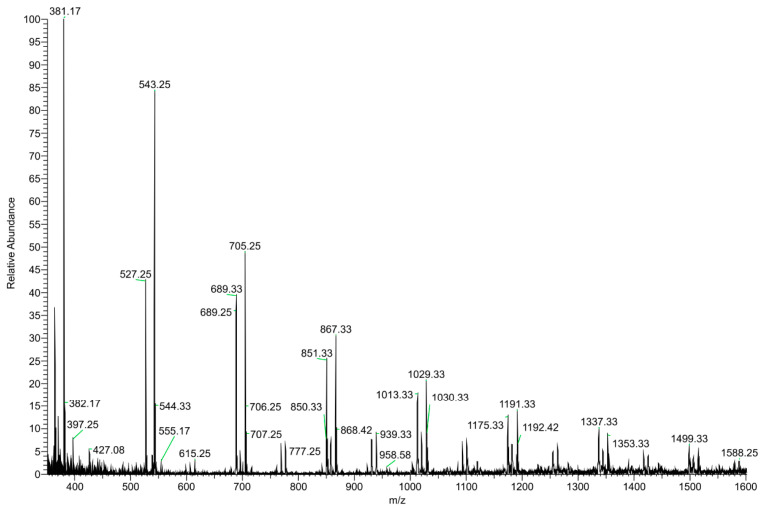
(+) ESI–MS full scan of the dry residue of garlic water extract (GWE).

**Figure 3:**
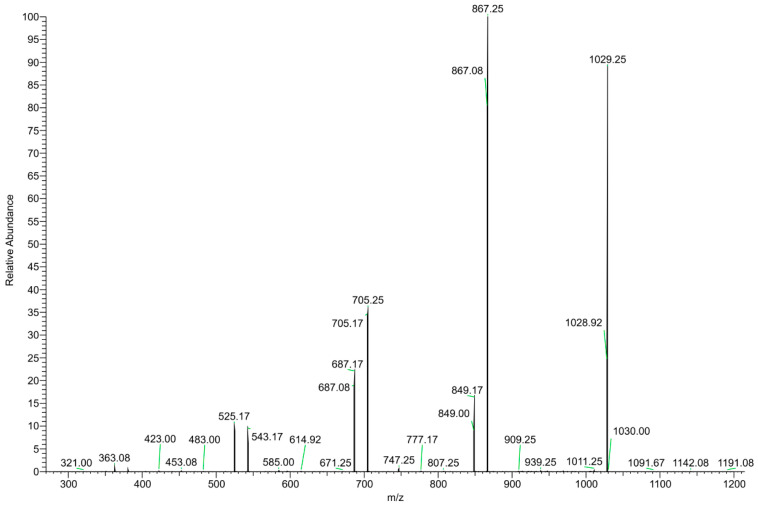
MS/MS spectrum of the *m/z* 1029 positively charged ion.

The DI–ESI–MS full spectrum was dominated by the regular repetition of two monocharged species differing by 16 mass units (Figure 2), that suggests the presence of species complexing Na^+^ and K^+^ cations. The 162 Da intervals correspond to the difference of one -C_6_H_10_O_5_- unit, typical of a homologous series of polysaccharides. The MS/MS spectrum of the 1029 *m/z* ion, reported in Figure 3 as representative ion of the series, showed the sequential loss of 162 Da (=C_6_H_10_O_5_) and 18 Da (=H_2_O) according to the MS2 fragmentation of a polysaccharide. The detected oligosaccharides, most probably fructans [41], are characterized by a wide degree of polymerization (DP), namely in the 2-11 DP range (Table 3). The Na^+^ and K^+^ ions distribution emerging from the fructans series indicates a larger content of the latter, despite what one should expect considering the ubiquitous presence of Na^+^ cation in the ESI–MS experiments. In addition to the fructans, the full scan showed two intense signals at *m*/*z* 175 and 214, corresponding to the protonated L-arginine and to N-butylbenzene sulfonamide (a plasticizer contaminant), respectively (Table 3).

#### 3.1.3. Colorimetric Analysis

**Figure 4:**
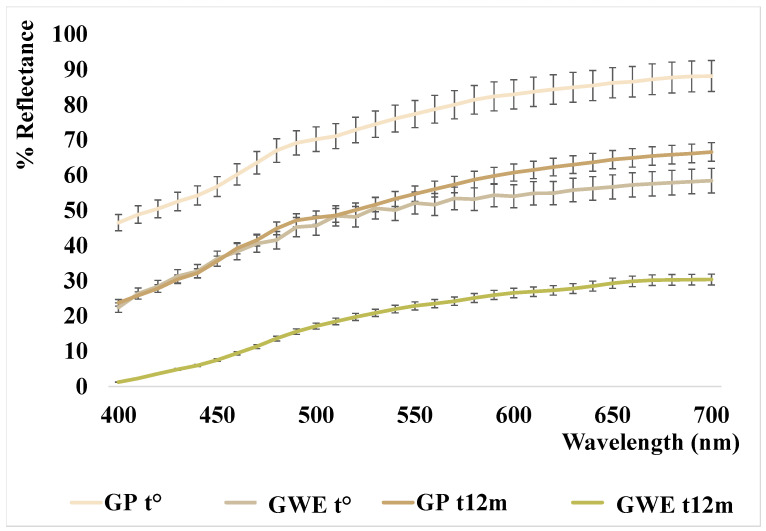
Reflectance curves of the analyzed garlic samples.

The color parameters and relative reflectance curves of the analyzed samples are shown in Table 4 and Figure 4.

As reported in our previous work [22], the garlic powder analyzed at t° has a very bright color tending to pale yellow (L* = 90.15 and b* = 16.02) that, after eight months of storage, seemed to fade slightly (L* ≈ 94 vs. 90 at t^0^, b* ≈ 14 vs. 16 at t^0^, with a ΔE ≈ 2, data previously reported). In this study, the analyses performed at t^12m^, showed a substantial change in color (ΔE = 12.34), characterized by a strong darkening of the powder (L* ≈ 78; b* ≈ 20), as also shown by the lowering of the reflectance curve reported in Figure 4. The initial light bleaching and subsequent browning were already described in milk powder samples in Cesa and collaborators (2015) [42], and interpreted as carotenoid degradation followed by Maillard reaction. In the case of garlic, bleaching was not previously described. Nevertheless, the presence of carotenoids in *Allium* spp. phytocomplex was reported in the literature [43] and the carotenoid bleaching seems the simplest hypothesis, despite all our attempts to extract a carotenoid fraction by organic solvents failed. Therefore, the slight bleaching should be attributed to other yellow, hydrosoluble, components. On the other hand, the powder darkening could be mainly due to the different sulfur components (the presence of many different sulfur compounds was confirmed by HS–SPME–GC–MS analysis (see Section 3.1.2)) which, modifying the pH and the activity of polyphenol oxidases, with a not completely known mechanism depending on the state of preservation and on the temperature, cause darkening of the matrix [44,45].

This darkening is also confirmed in the aqueous extract newly prepared and analyzed after 12 months (see Figure 4). In fact, the L* parameter of GWE t° decreases significantly from 76 to 65, as well as the b* parameter increases even more, from 17 to 28, denoting altogether a strong color change characterized by a substantial browning with respect to the starting points (ΔE = 28 respect to G_P_ t° and ΔE = 16 respect to GWE t°).

#### 3.1.4. HPLC–DAD Analysis

The HPLC–DAD analysis was conducted at 254 nm for the identifying benzoic and hydroxycinnamic acids, flavanols and organosulfur compounds. The molecular profile is confirmed by literature even if no chromatograms of aqueous extracts are reported [46,47].

The chromatogram showed the presence of alliin and an its diastereoisomer (361.1 ± 17.3 µg/g dry extract) as reported by Dethier et al., 2012 [48]. Furthermore, chlorogenic acid, caffeic acid, and epicatechin (see Section 3.1.1 for the relevant quantification) were also shown.

These results are only partially comparable to those present in the literature, because the garlic phytocomplex and relative aqueous extracts are very variable according to the different cultivar, geographic area, and storage conditions. There are no references in the literature on quantitative data related to garlic aqueous extracts. Existing data refer to methanolic or hydroalcoholic extracts reporting alliin content in range of 0.5–33 expressed in mg/g dry weight [47,49]. Compared to the hydroalcoholic extract, reported in our previous work [22], alliin values found in GWE are significantly lower (approximately 360 vs. 1200 μg/g dry extract).

### 3.2. Toxicological and Pharmacological Studies

**Figure 5:**
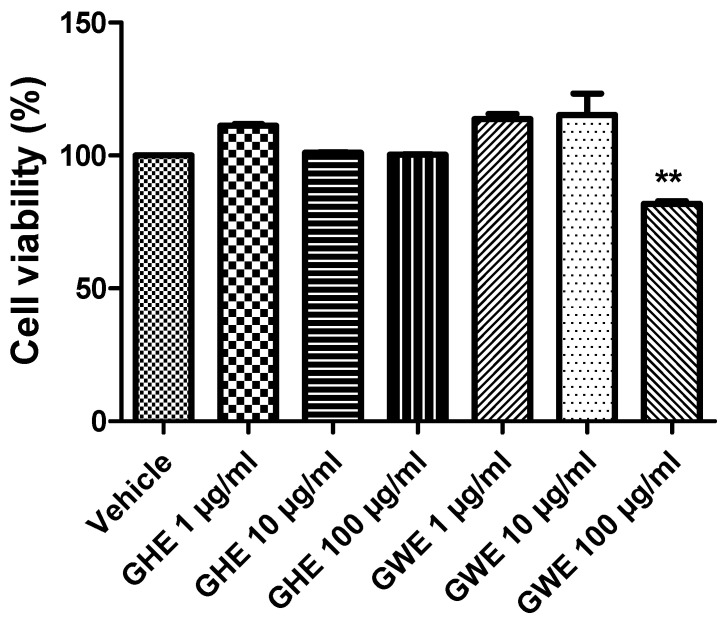
MTT assay of SW480 cell line treated with garlic hydroalcoholic extract (GHE) (1, 10, and 100 μg/mL), garlic water extract (GWE) (1, 10, and 100 μg/mL), and vehicle (RPMI) for 48 h, in basal conditions. Data are displayed as the means ± SEM. ANOVA, *p* < 0.001; ** *p* < 0.01 vs. vehicle.

In a previous study of ours, GHE (1–100 μg/mL) was not able to modify cell viability of H9c2 cells (rat cardiomyoblasts), in basal conditions, confirming its good biocompatibility [22]. Moreover, the same extract (10–100 μg/mL) was effective in protecting cells from cytotoxicity induced by H_2_O_2_ (200 μM) [22]. In the present study, we investigated the effects of GHE and GWE, in the dose range 1–100 μg/mL, on colon cancer SW480 cell line viability, in basal conditions. Compared to the control group, GHE was not able to affect SW480 cell viability (Figure 5). However, GWE (100 μg/mL) significantly suppressed SW480 cell viability, even if the cell viability was not under the biocompatibility limits (70% viability compared to control, respectively) (Figure 5).

**Figure 6:**
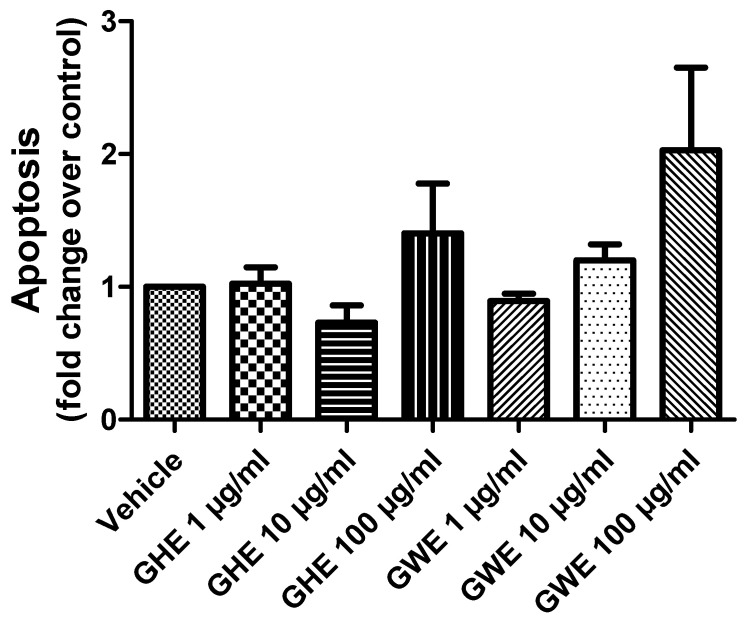
Apoptosis assay in SW480 cell line treated with garlic hydroalcoholic extract (GHE) (1, 10, and 100 μg/mL), garlic water extract (GWE) (1, 10, and 100 μg/mL), and vehicle (RPMI) for 48 h, in basal conditions.

Accordingly, both GHE and GWE were not able to modify apoptosis of SW480 cell lines following 48 h of treatment in basal conditions (Figure 6).

**Figure 7:**
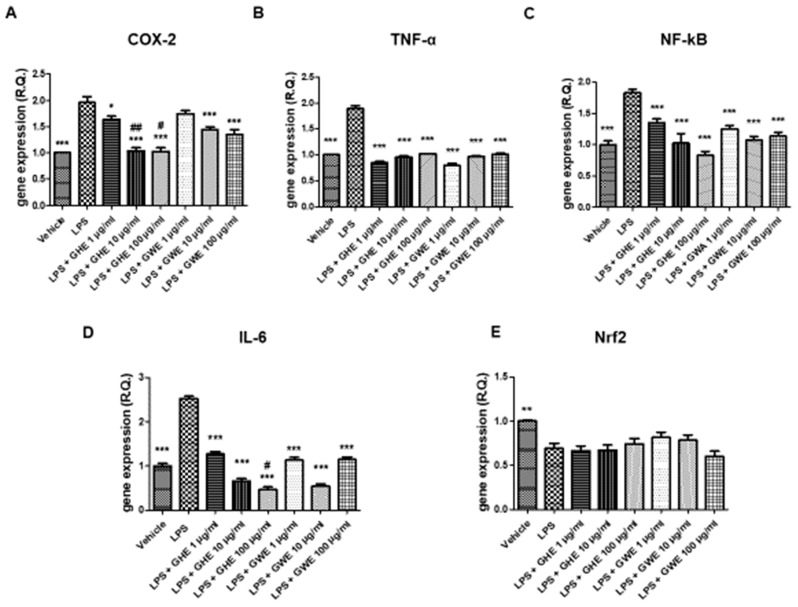
Effects of garlic hydroalcoholic extract (GHE) (1, 10, and 100 μg/mL), garlic water extract (GWE) (1, 10, and 100 μg/mL) and vehicle (RPMI) on LPS-induced cyclooxygenase (COX)-2 (**A**), tumor necrosis factor (TNF)-α (**B**) nuclear factor-kB (NF-kB (**C**)), interleukin (IL)-6 (**D**), nuclear factor erythroid 2-related factor 2 (Nrf2) (**E**) gene expression (RQ, relative quantification) in mouse colon specimens. Data are displayed as the means ± SEM. ANOVA, *p* < 0.01; * *p* < 0.05, ** *p* < 0.01 vs. LPS, and *** *p* < 0.001 vs. LPS; ^#^ *p* < 0.05 vs. LPS+GWE 100 μg/mL; ^##^ *p* < 0.01 vs. GWE 10 μg/mL.

GHE and GWE, in the dose range 1–100 μg/mL, were then tested to evaluate their potential protective activities on oxidative and inflammation pathways in mouse colon specimens treated with LPS. In particular, GHE, in the tested dose range, was effective in suppressing LPS-induced gene expression of pro-inflammatory markers strongly involved in colon inflammation, including COX-2, TNF-α, NF-κB, and IL-6 (Figure 7A–E) [50,51,52,53]. In the same experimental paradigm, GWE (1–100 μg/mL) inhibited LPS-induced TNF-α, NF-κB, and IL-6 (Figure 7A–E) gene expression. Moreover, the higher concentrations of GWE suppressed COX-2 (Figure 7A) gene expression induced by LPS treatment. On the other hand, GHE and GWE did not modify LPS-induced Nrf2 gene expression (Figure 7E), ruling out a possible role of this mediator in mediating the protective effects exerted by GWE and GHE in mouse colon.

**Figure 8:**
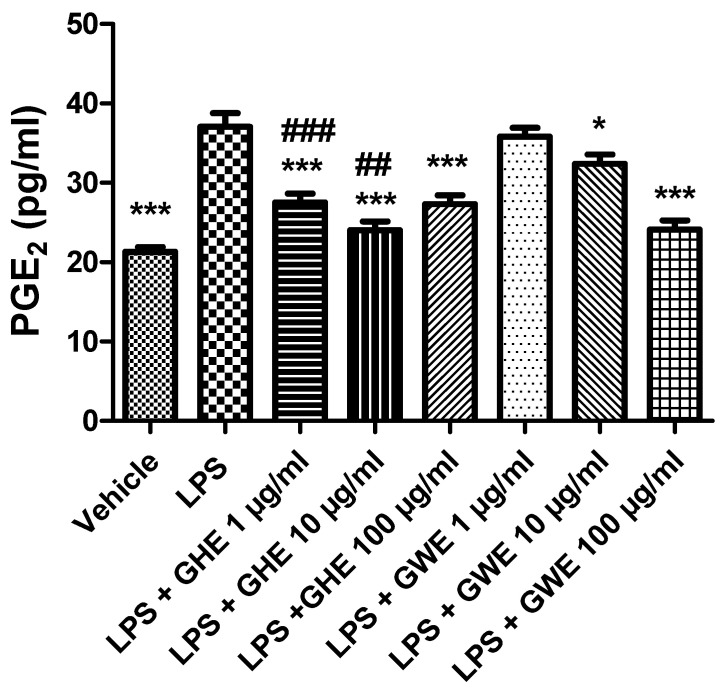
Effects of garlic hydroalcoholic extract (GHE) (1, 10, and 100 μg/mL), garlic water extract (GWE) (1, 10, and 100 μg/mL) and vehicle (RPMI) on LPS-induced prostaglandin E_2_ (PGE_2_) levels in mouse colon specimens. Data are shown as the means ± SEM. ANOVA, *p* < 0.0001; * *p* < 0.05, and *** *p* < 0.001 vs. LPS; ^##^ *p* < 0.01, ^###^ *p* < 0.001 vs. co-respective treatment with GWE.

Accordingly, Hodge and collaborators (2002) [54] showed that, in peripheral blood monocytes, production of various leukocyte pro-inflammatory cytokines, such as IL-6, IL-8 and TNF-α, was reduced by a garlic extract (≥10.0 µg/mL). On the other hand, it also stimulated IL-10 synthesis in the same experimental model. In this context, the same authors suggested a possible therapeutic use of garlic in the management of inflammatory conditions, including inflammatory bowel disease [54]. Our present findings also agree with those by Shin and collaborators (2013) [55], who observed inhibitory effects induced by fresh and heated raw garlic extracts (FRGE and HRGE) on LPS-induced release of pro-inflammatory cytokines, such as IL-1β, TNF-α, and IL-6, in RAW 264.7 macrophages. Moreover, we investigated the effects of the extracts on LPS-induced levels of PGE_2_, a pro-inflammatory mediator generated by COX-2, which is strongly involved in the pathophysiology of inflammatory bowel disease [56]. Our present results showed that both GHE (1–100 μg/mL) and GWE (10–100 μg/mL) suppressed LPS-induced PGE_2_ levels in isolated colon specimens (Figure 8), further supporting the anti-inflammatory effects exerted by these extracts.

**Figure 9:**
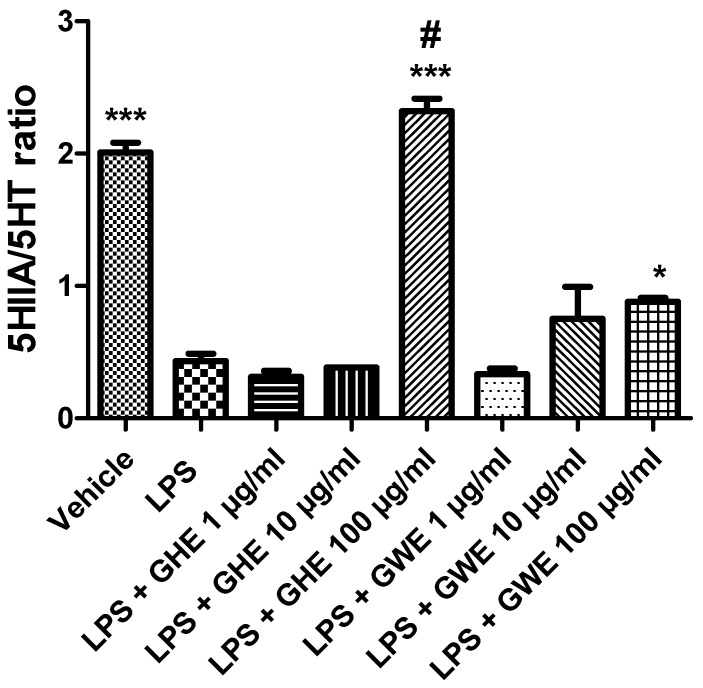
Effects of garlic hydroalcoholic extract (GHE) (1, 10, and 100 μg/mL), garlic water extract (GWE) (1, 10, and 100 μg/mL) and vehicle (RPMI) on the 5-HIIA/5-HT ratio in mouse colon specimens treated with vehicle. Data are displayed as the means ± SEM. ANOVA, *p* < 0.0001; * *p* < 0.05, and *** *p* < 0.001 vs. LPS; ^#^ *p* < 0.001 vs. LPS + GWE 100 μg/mL.

Accordingly, we previously reported that GWE exerted cardioprotective activities, by suppressing LPS-induced COX-2, IL-6, and NF-kB gene expression, as well as PGE_2_ levels, in heart specimens. To this regard, we hypothesized that the suppression of NF-*k*B gene expression could be involved in modulating the inhibitory effects induced by GWE on COX-2 gene expression and PGE_2_ production [22]. Accordingly, ethyl linoleate from garlic was reported to suppress LPS-induced COX-2 mRNA and protein expression as well as PGE_2_ production in RAW264.7 cells, by suppressing NF-*k*B activation [57]. Actually, the anti-inflammatory effects exerted by GHE and GWE could be related, at least in part, to their phenol and flavonoid content [58,59,60,61,62,63,64,65,66,67], with particular regards to catechin [58]. To this regard, catechin was suggested to act as a potential therapeutic agent in the prevention of inflammation. In particular, catechin exhibited anti-inflammatory properties by suppressing inducible nitric oxide synthase (iNOS), and COX-2 protein expression, as well as IL-6 and TNF-α mRNA levels in LPS-treated RAW264.7 cells [68]. Interestingly, GHE was shown more effective than GWE in decreasing gene expression of COX-2, and IL-6 (Figure 7A,D), as well as PGE_2_ levels (Figure 9) in isolated mouse colon specimens following LPS challenge. This finding could be related to its major content in catechin [69], resveratrol [70], naringenin [71], and hesperetin [72]. Accumulating evidence showed that 5-HT is a pro-inflammatory mediator critically involved in the pathogenesis of intestinal disorders, such as IBD [73,74,75], irritable bowel syndrome [73,76,77], as well as DSS-induced experimental colitis [78]. In the present study, we also investigated the effects of the garlic extracts on the LPS-induced 5HIIA/5-HT ratio, which is known as a useful index of 5-HT turnover, in vivo [79,80], deeply related to the activity of monoamine oxidase (MAO)-A [81].

Consistently with the literature data [33], we found that LPS reduced the 5HIIA/5-HT ratio (Figure 9) in isolated mouse colon specimens.

However, GHE and GWE (100 μg/mL) prevented LPS-induced reduction in 5-HT turnover. The decreased levels of 5-HT, measured as the 5HIIA/5-HT ratio, could further account for the anti-inflammatory effects exerted by garlic extracts. To this regard, we have previously found that anti-inflammatory herbal extracts suppressed 5-HT levels in isolated rat colon treated with LPS [82,83]. In particular, GHE was found more effective than GWE in counteracting the LPS-induced decrease in the 5HIIA/5-HT ratio (Figure 9). Actually, the higher activity of GHE compared to GWE could be related to its higher content in benzoic acid and flavonoids, such as quercetin [84,85].

**Figure 10:**
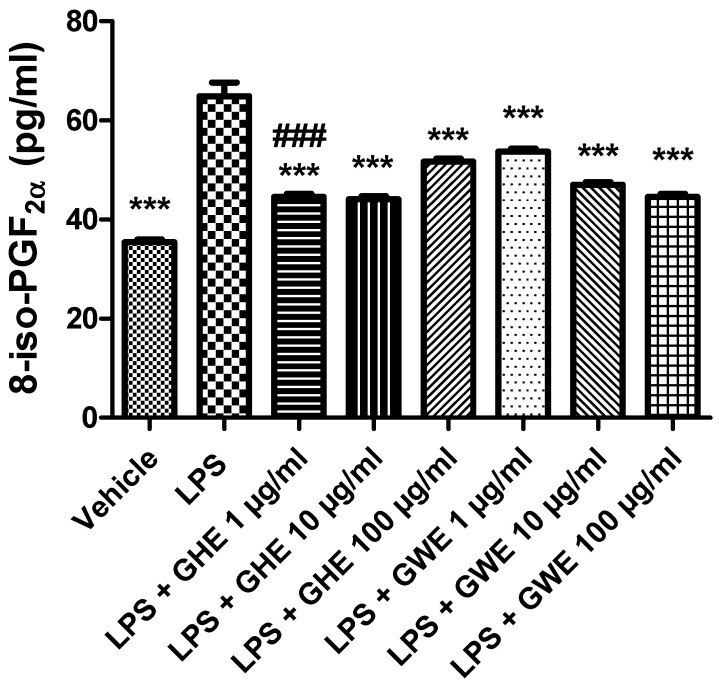
Effects of garlic hydroalcoholic extract (GHE) (1, 10, and 100 μg/mL), garlic water extract (GWE) (1, 10, and 100 μg/mL) and vehicle (RPMI) on LPS-induced 8-iso-prostaglandin (PG)F_2α_ levels in mouse colon specimens. Data are shown as the means ± SEM. ANOVA, *p* < 0.0001; *** *p* < 0.001 vs. LPS; ^###^ *p* < 0.001 vs. co-respective treatment with GWE.

A wide body of evidence showed that imbalance between the oxidative reactions and antioxidant defense mechanisms played a key role in the initiation and progression of IBD. This imbalance generates oxidative stress resulting from either reactive oxygen species (ROS) overproduction or a reduction in antioxidant activity [86,87]. In particular, ROS overproduction was suggested to be involved in functional disruption of the enteric mucosa [52]. Increased production of ROS is known to damage cellular lipids, proteins, as well as nucleic acids, and finally disrupt gastrointestinal barrier integrity [88]. 8-iso-PGF_2α_ is an isomer of prostaglandins produced from membrane arachidonic acid by free radical-catalyzed peroxidation, which is regarded as a stable marker of lipid peroxidation and oxidative stress [89]. As shown in Figure 10, both GHE and GWE (1–100 μg/mL) were able to counteract 8-iso-PGF_2α_ levels induced by LPS treatment in isolated mouse colon specimens. In particular, GHE (1 μg/mL) showed higher efficacy in inhibiting 8-iso-PGF_2α_ levels (Figure 10).

These results agreed with the antioxidant effects induced by GHE, tested in the same concentration range, in isolated mouse heart [22]. Accordingly, aqueous garlic extract was also shown to possess antioxidant properties by scavenging ROS and increasing cellular antioxidant enzymes including superoxide dismutase, catalase, and glutathione peroxidase [36]. The antioxidant effects induced by GHE and GWE are consistent with their polyphenol content [90], with particular regards to catechin [58]. In this context, catechins were found able to reduce the colonic oxidative damages to the colon, by suppressing oxidative stress, via exerting direct or indirect antioxidant effects, and by enhancing the activity of various antioxidant enzymes including glutathione peroxidases [58].

## 4. Conclusions

Concluding, GHE and GWE, particularly GHE, showed protective effects, as confirmed by the inhibitory effects on selected pro-inflammatory and pro-oxidant markers, in LPS-stimulated colon, suggesting a potential role in the prevention and management of ulcerative colitis. The phytochemical analyses suggested these effects could be related, albeit partially, to their phenol and flavonoid content, with particular regards to catechin. Moreover, other components of nutraceutical and pharmaceutical interest were detected in these extracts. On the other hand, further studies are necessary to accurately evaluate the in vivo activity.

## Figures and Tables

**Table 1:** Quantification of the investigated phenolic compounds detected in garlic hydroalcoholic extract (GHE) and garlic water extract (GWE) (μg/mL).

Compound	GHE	GWE
Gallic acid	17.21 ± 0.17	16.88 ± 0.12
3-Hydroxytyrosol	10.13 ± 0.15 ***	12.86 ± 0.22
Caftaric acid	0.93 ± 0.06	n.d.
Catechin	37.40 ± 1.25 ***	21.38 ± 0.72
4-Hydroxybenzoic acid	n.d.	n.d.
Loganic acid	n.d.	n.d.
Chlorogenic acid	13.54 ± 0.72	12.14 ± 0.53
Vanillic acid	n.d.	n.d.
Caffeic acid	10.91 ± 0.95	10.19 ± 0.58
Epicatechin	8.94 ± 0.23 *	7.23 ± 0.40
Syringaldehyde	9.53 ± 0.12	9.54 ± 0.23
*p*-Coumaric acid	13.33 ± 0.17	12.73 ± 0.15
*t*-Ferulic acid	15.82 ± 0.26 **	13.43 ± 0.21
Benzoic acid	22.66 ± 0.35 *	21.51 ± 0.18
Rutin	13.68 ± 0.22	13.08 ± 0.23
Resveratrol	16.18 ± 0.27 **	13.97 ± 0.15
*t*-Cinnamic acid	11.83 ± 0.09	11.59 ± 0.12
Quercetin	15.86 ± 0.20 *	14.76 ± 0.21
Naringenin	17.93 ± 0.21 *	16.02 ± 0.37
Hesperetin	17.06 ± 0.58 **	12.72 ± 0.31
Kaempferol	n.d.	n.d.
Carvacrol	n.d.	n.d.
Thymol	n.d.	n.d.
Flavone	20.43 ± 0.15	19.83 ± 0.19
3-Hydroxyflavone	10.27 ± 0.29 **	13.46 ± 0.25
Emodin	n.d.	n.d.

Values expressed are the means ± S.D. of three parallel measurements. n.d., not detected. *t*-test, * *p* < 0.05, ** *p* < 0.01, and *** *p* < 0.001 vs. vehicle.

**Table 2:** HS–SPME–GC–MS analysis of dry residue of garlic water extract (GWE).

Compound	Class	Area%	RI	RI_L_^a^
2-Propenal ^b^	Aldehyde	1.89		
Heptane ^b^	Other	0.33		
Diallyl sulfide	SCC	0.43	864	857
Methyl allyl disulfide	SCC	1.47	927	919
Methoxy phenyl oxime ^b^	Other	5.72	943	
*p*-cymene	Monoterpene	0.47	1041	1025
Allyl disulfide	SCC	9.52	1088	1090
3-Allyl-thio-propionic acid	SCC	1.70	1107	1093
Linalyl anthranilate	Monoterpenoid	0.95	1110	1104
Nonanal	Aldehyde	1.16	1113	1102
Methyl 2-propenyl trisulfide	SCC	2.51	1146	1142
Isoborneol	Monoterpenoid	0.44	1178	1165
Terpinen-4-ol	Monoterpenoid	0.55	1187	1184
3-Vinyl-1,2-dithiacyclohex-4-ene	SCC	3.18	1195	1191
Alpha-terpineol	Monoterpenoid	0.27	1203	1207
Decanal	Aldehyde	0.63	1214	1208
3-Vinyl-1,2-dithiacyclohex-5-ene	SCC	3.96	1222	1224
Thymol	Monoterpenoid	22.4	1310	1293
Carvacrol	Monoterpenoid	36.1	1319	1317
7 unknown compounds		6.32		
**Class**				
Monoterpene and derivatives		61.2		
SCC		22.8		
Aldehyde		3.68		
Other		6.05		

^a^ RI reported in literature; ^b^ MS-only identification method.

**Table 3:** DI–ESI–MS analysis of dry residue of garlic water extract (GWE).

*m/z*	Compound		Charge
Positive mode			
163	Allicin		H^+^
175	Arginine		H^+^
214	N-butylbenzene sulfonamide		H^+^
		**DP**	
365	Disaccharide		Na^+^
381	Disaccharide		K^+^
527	Trisaccharide	3	Na^+^
543	Trisaccharide	3	K^+^
689	Oligosaccharide	4	Na^+^
705	Oligosaccharide	4	K^+^
851	Oligosaccharide	5	Na^+^
867	Oligosaccharide	5	K^+^
1013	Oligosaccharide	6	Na^+^
1029	Oligosaccharide	6	K^+^
1175	Oligosaccharide	7	Na^+^
1191	Oligosaccharide	7	K^+^
1337	Oligosaccharide	8	Na^+^
1353	Oligosaccharide	8	K^+^
1449	Oligosaccharide	9	Na^+^
1515	Oligosaccharide	9	K^+^
1661	Oligosaccharide	10	Na^+^
1677	Oligosaccharide	10	K^+^
1823	Oligosaccharide	11	Na^+^
1839	Oligosaccharide	11	K^+^
*Negative mode*			
191	Citric acid		[M-H]^−^
289	Catechin		[M-H]^−^

DP: degree of polymerization.

**Table 4:** Colorimetric CIEL*a*b* parameters of garlic powder (GP) and garlic water extract (GWE).

	L*	a*	b*	c*	h°	DE°
**GP t°**	90.15	0.47	16.02	16.03	88.32	-
**GP t^12m^**	78.55	1.11	20.17	20.20	86.85	12.34 ^a^
**GWE t°**	76.41	−1.10	16.83	16.87	93.74	13.85 ^a^
**GWE t^12m^**	65.00	−2.87	28.00	27.95	95.90	28.06 ^a^–16.06 ^b^

Reported values represent the mean of four measurements. Mean error < 2%. ΔE represents the overall color variation, ^a^ using GP t° as reference, and ^b^ using GWE t° as reference ΔE = [(L*_2_–L*_1_)^2^ + (a*_2_–a*_1_)^2^ + (b*_2_–b*_1_) ^2^]^½^.

## Data Availability

The data presented in this study are available on request from the corresponding author.

## Supplementary Materials

The following supporting information can be downloaded at: https://www.mdpi.com/article/10.3390/foods11223559/s1. Table S1: MS analysis conditions. Table S2: Retention times, wavelengths of quantification, mass to charge (*m*/*z*) ratios, and molecular weight of the investigated phenolic compounds. Table S3: Gradient elution conditions of the HPLC-DAD analyses for investigated polyphenolic compounds in garlic hydroalcoholic and water extracts (GHE and GWE).

## References

[1] Shang A., Cao S.Y., Xu X.Y., Gan R.Y., Tang G.Y., Corke H., Mavumengwana V., Li H.B. (2019). Bioactive Compounds and Biological Functions of Garlic (*Allium sativum* L.). Foods.

[2] Gu C., Howell K., Dunshea F.R., Suleria H.A. (2019). LC-ESI-QTOF/MS characterisation of phenolic acids and flavonoids in polyphenol-rich fruits and vegetables and their potential antioxidant activities. Antioxidants.

[3] Diretto G., Rubio-Moraga A., Argandoña J., Castillo P., Gómez-Gómez L., Ahrazem O. (2017). Tissue-specific accumulation of sulfur compounds and saponins in different parts of garlic cloves from purple and white ecotypes. Molecules.

[4] Wang Y., Guan M., Zhao X., Li X. (2018). Effects of garlic polysaccharide on alcoholic liver fibrosis and intestinal microflora in mice. Pharm. Biol..

[5] Beato V.M., Orgaz F., Mansilla F., Montaño A. (2011). Changes in phenolic compounds in garlic (*Allium sativum* L.) owing to the cultivar and location of growth. Plant Foods Hum. Nutr..

[6] Waterer D., Schmitz D. (1994). Influence of variety and cultural practices on garlic yields in Saskatchewan. Can. J. Plant Sci..

[7] Locatelli D.A., Nazareno M.A., Fusari C., Camargo A. (2017). Cooked garlic and antioxidant activity: Correlation with organosulfur compound composition. Food Chem..

[8] Batiha G.E., Beshbishy A.M., Wasef L.G., Elewa Y.H.A., Al-Sagan A.A., El-Hack M.E.A., Taha A.E., Abd-Elhakim Y.M., Devkota H.P. (2020). Chemical Constituents and Pharmacological Activities of Garlic (*Allium sativum* L.): A Review. Nutrients.

[9] Koutroubakis I.E., Malliaraki N., Dimoulios P.D., Karmiris K., Castanas E., Kouroumalis E.A. (2004). Decreased Total and Corrected Antioxidant Capacity in Patients with Inflammatory Bowel Disease. Am. J. Dig. Dis..

[10] Rezaie A., Parker R.D., Abdollahi M. (2007). Oxidative Stress and Pathogenesis of Inflammatory Bowel Disease: An Epiphenomenon or the Cause?. Dig. Dis. Sci..

[11] Achitei D., Ciobica A., Balan G., Gologan E., Stanciu C., Stefanescu G. (2013). Different Profile of Peripheral Antioxidant Enzymes and Lipid Peroxidation in Active and Non-active Inflammatory Bowel Disease Patients. Am. J. Dig. Dis..

[12] Chung H.-L., Yue G.G.-L., To K.-F., Su Y.-L., Huang Y., Ko W.-H. (2007). Effect of Scutellariae Radix extract on experimental dextran-sulfate sodium-induced colitis in rats. World J. Gastroenterol..

[13] Lenoir L., Joubert-Zakeyh J., Texier O., Lamaison J.-L., Vasson M.-P., Felgines C. (2011). Aloysia triphylla infusion protects rats against dextran sulfate sodium-induced colonic damage. J. Sci. Food Agric..

[14] Harisa G.E., Abo-Salem O.M., El-Sayed E.-S.M., Taha E.I., El-Halawany N. (2009). L-arginine augments the antioxidant effect of garlic against acetic acid-induced ulcerative colitis in rats. Pak. J. Pharm. Sci..

[15] Kuo C.H., Lee S.H., Chen K.M., Lii C.K., Liu C.T. (2011). Effect of garlic oil on neutrophil infiltration in the small intestine of endotoxin-injected rats and its association with levels of soluble and cellular adhesion molecules. J. Agric. Food Chem..

[16] Balaha M., Kandeel S., Elwan W. (2016). Garlic oil inhibits dextran sodium sulfate-induced ulcerative colitis in rats. Life Sci..

[17] Tanrıkulu Y., Şen Tanrıkulu C., Kılınç F., Can M., Köktürk F. (2020). Effects of garlic oil (allium sativum) on acetic acid-induced colitis in rats: Garlic oil and experimental colitis. Turk. J. Trauma Emerg. Surg..

[18] Recinella L., Chiavaroli A., Ronci M., Menghini L., Brunetti L., Leone S., Tirillini B., Angelini P., Covino S., Venanzoni R. (2020). Multidirectional Pharma-Toxicological Study on Harpagophytum procumbens DC. ex Meisn.: An IBD-Focused Investigation. Antioxidants.

[19] Vadalà R., Mottese A.F., Bua G.D., Salvo A., Mallamace D., Corsaro C., Vasi S., Giofrè S.V., Alfa M., Cicero N. (2016). Statistical Analysis of Mineral Concentration for the Geographic Identification of Garlic Samples from Sicily (Italy), Tunisia and Spain. Foods.

[20] Fujisawa H., Suma K., Origuchi K., Seki T., Ariga T. (2008). Thermostability of allicin determined by chemical and biological assays. Biosci. Biotechnol. Biochem..

[21] Fujisawa H., Suma K., Origuchi K., Kumagai H., Seki T., Ariga T. (2008). Biological and chemical stability of garlic-derived allicin. J. Agric. Food Chem..

[22] Recinella L., Chiavaroli A., Masciulli F., Fraschetti C., Filippi A., Cesa S., Cairone F., Gorica E., De Leo M., Braca A. (2021). Protective Effects Induced by a Hydroalcoholic Allium sativum Extract in Isolated Mouse Heart. Nutrients.

[23] Chiavaroli A., Balaha M., Acquaviva A., Ferrante C., Cataldi A., Menghini L., Rapino M., Orlando G., Brunetti L., Leone S. (2021). Phenolic Characterization and Neuroprotective Properties of Grape Pomace Extracts. Molecules.

[24] Florio R., De Lellis L., Veschi S., Verginelli F., di Giacomo V., Gallorini M., Perconti S., Sanna M., Mariani-Costantini R., Natale A. (2018). Effects of dichloroacetate as single agent or in combination with GW6471 and metformin in paraganglioma cells. Sci. Rep..

[25] Veschi S., De Lellis L., Florio R., Lanuti P., Massucci A., Tinari N., De Tursi M., di Sebastiano P., Marchisio M., Natoli C. (2018). Effects of repurposed drug candidates nitroxoline and nelfinavir as single agents or in combination with erlotinib in pancreatic cancer cells. J. Exp. Clin. Cancer Res..

[26] Recinella L., Chiavaroli A., Orlando G., Menghini L., Ferrante C., Di Cesare Mannelli L., Ghelardini C., Brunetti L., Leone S. (2019). Protective Effects Induced by Two Polyphenolic Liquid Complexes from Olive (Olea europaea, mainly Cultivar Coratina) Pressing Juice in Rat Isolated Tissues Challenged with LPS. Molecules.

[27] Recinella L., Micheli L., Chiavaroli A., Libero M.L., Orlando G., Menghini L., Acquaviva A., Di Simone S., Ferrante C., Ghelardini C. (2022). Anti-Inflammatory Effects Induced by a Polyphenolic Granular Complex from Olive (Olea europaea, Mainly Cultivar coratina): Results from In Vivo and Ex Vivo Studies in a Model of Inflammation and MIA-Induced Osteoarthritis. Nutrients.

[28] Recinella L., Chiavaroli A., Orlando G., Ferrante C., Marconi G.D., Gesmundo I., Granata R., Cai R., Sha W., Schally A.V. (2020). Antinflammatory, antioxidant, and behavioral effects induced by administration of growth hormone-releasing hormone analogs in mice. Sci. Rep..

[29] Recinella L., Chiavaroli A., Di Valerio V., Veschi S., Orlando G., Ferrante C., Gesmundo I., Granata R., Cai R., Sha W. (2021). Protective effects of growth hormone-releasing hormone analogs in DSS-induced colitis in mice. Sci. Rep..

[30] Recinella L., Chiavaroli A., Orlando G., Ferrante C., Veschi S., Cama A., Marconi G.D., Diomede F., Gesmundo I., Granata R. (2021). Effects of growth hormone-releasing hormone receptor antagonist MIA-602 in mice with emotional disorders: A potential treatment for PTSD. Mol. Psychiatry.

[31] Recinella L., Chiavaroli A., Veschi S., Di Valerio V., Lattanzio R., Orlando G., Ferrante C., Gesmundo I., Granata R., Cai R. (2022). Antagonist of growth hormone-releasing hormone MIA-690 attenuates the progression and inhibits growth of colorectal cancer in mice. Biomed. Pharmacother..

[32] Livak K.J., Schmittgen T.D. (2001). Analysis of relative gene expression data using real-time quantitative PCR and the 2(-Delta Delta C(T)) Method. Methods.

[33] Zengin G., Locatelli M., Ferrante C., Menghini L., Orlando G., Brunetti L., Recinella L., Chiavaroli A., Leone S., Leporini L. (2019). New pharmacological targets of three Asphodeline species using in vitro and ex vivo models of inflammation and oxidative stress. Int. J. Environ. Health Res..

[34] Orlando G., Leone S., Ferrante C., Chiavaroli A., Mollica A., Stefanucci A., Macedonio G., Dimmito M.P., Leporini L., Menghini L. (2018). Effects of Kisspeptin-10 on Hypothalamic Neuropeptides and Neurotransmitters Involved in Appetite Control. Molecules.

[35] Charan J., Kantharia N.D. (2013). How to calculate sample size in animal studies?. J. Pharmacol. Pharmacother..

[36] Arreola R., Quintero-Fabián S., López-Roa R.I., Flores-Gutiérrez E.O., Reyes-Grajeda J.P., Carrera-Quintanar L., Ortuño-Sahagún D. (2015). Immunomodulation and anti-inflammatory effects of garlic compounds. J. Immunol. Res..

[37] Sivam G.P. (2001). Protection against Helicobacter pylori and other bacterial infections by garlic. J. Nutr..

[38] Melguizo-Rodríguez L., García-Recio E., Ruiz C., De Luna-Bertos E., Illescas-Montes R., Costela-Ruiz V.J. (2022). Biological properties and therapeutic applications of garlic and its components. Food Funct..

[39] Bozin B., Mimica-Dukic N., Samojlik I., Goran A., Igic R. (2008). Phenolics as antioxidants in garlic (*Allium sativum* L., Alliaceae). Food Chem..

[40] Ramirez D.A., Altamirano J.C., Camargo A.B. (2021). Multi-phytochemical determination of polar and non-polar garlic bioactive compounds in different food and nutraceutical preparations. Food Chem..

[41] Lu X., Li N., Zhao R., Zhao M., Cui X., Xu Y., Qiao X. (2021). In vitro Prebiotic Properties of Garlic Polysaccharides and Its Oligosaccharide Mixtures Obtained by Acid Hydrolysis. Front. Nutr..

[42] Cesa S., Casadei M.A., Cerreto F., Paolicelli P. (2015). Infant milk formulas: Effect of storage conditions on the stability of powdered products towards autoxidation. Foods.

[43] Tuan P.A., Kim J.K., Kim H.H., Lee S.Y., Park N.I., Park S.U. (2011). Carotenoid accumulation and characterization of cDNAs encoding phytoene synthase and phytoene desaturase in garlic (*Allium sativum*). J. Agric. Food Chem..

[44] Hong S.I., Kim D.M. (2001). Storage quality of chopped garlic as influenced by organic acids and high-pressure treatment. J. Sci. Food Agric..

[45] Liu P., Lu X., Li N., Zheng Z., Zhao R., Tang X., Qiao X. (2019). Effects and mechanism of free amino acids on browning in the processing of black garlic. J. Sci. Food Agric..

[46] Ichikawa M., Ryu K., Yoshida J., Ide N., Kodera Y., Sasaoka T., Rosen R.T. (2003). Identification of six phenylpropanoids from garlic skin as major antioxidants. J. Agric. Food Chem..

[47] Eghdami A., Sohi S.M.H., Asli D.E., Houshmandfar A. (2011). Antioxidant activity of methanolic and hydroalcohlic extracts of garlic plant. Adv. Environ. Biol..

[48] Dethier B., Laloux M., Hanon E., Nott K., Heuskin S., Wathelet J.P. (2012). Analysis of the diastereoisomers of alliin by HPLC. Talanta.

[49] Bloem E., Haneklaus S., Schnug E. (2005). Influence of nitrogen and sulfur fertilization on the alliin content of onions and garlic. J. Plant Nutr..

[50] Atreya I., Atreya R., Neurath M.F. (2008). NF-kappaB in inflammatory bowel disease. J. Intern. Med..

[51] Bouguen G., Chevaux J.B., Peyrin-Biroulet L. (2011). Recent advances in cytokines: Therapeutic implications for inflammatory bowel diseases. World J. Gastroenterol..

[52] Biasi F., Astegiano M., Maina M., Leonarduzzi G., Poli G. (2011). Polyphenol supplementation as a complementary medicinal approach to treating inflammatory bowel disease. Curr. Med. Chem..

[53] Li Q., Verma I.M. (2002). NF-kB regulation in the immune system. Nat. Rev. Immunol..

[54] Hodge G., Hodge S., Han P. (2002). Allium sativum (garlic) suppresses leukocyte inflammatory cytokine production in vitro: Potential therapeutic use in the treatment of inflammatory bowel disease. Cytometry.

[55] Shin J.H., Ryu J.H., Kang M.J., Hwang C.R., Han J., Kang D. (2013). Short-term heating reduces the anti-inflammatory effects of fresh raw garlic extracts on the LPS-induced production of NO and pro-inflammatory cytokines by downregulating allicin activity in RAW 264.7 macrophages. Food Chem. Toxicol..

[56] Dey I., Lejeune M., Chadee K. (2006). Prostaglandin E2 receptor distribution and function in the gastrointestinal tract. Br. J. Pharmacol..

[57] Park S.Y., Seetharaman R., Ko M.J., Kim D.Y., Kim T.H., Yoon M.K., Kwak J.H., Lee S.J., Bae Y.S., Choi Y.W. (2014). Ethyl linoleate from garlic attenuates lipopolysaccharide-induced pro-inflammatory cytokine production by inducing heme oxygenase-1 in RAW264.7 cells. Int. Immunopharmacol..

[58] Fan F.Y., Sang L.X., Jiang M. (2017). Catechins and Their Therapeutic Benefits to Inflammatory Bowel Disease. Molecules.

[59] Bai J., Zhang Y., Tang C., Hou Y., Ai X., Chen X., Zhang Y., Wang X., Meng X. (2021). Gallic acid: Pharmacological activities and molecular mechanisms involved in inflammation-related diseases. Biomed. Pharmacother..

[60] Zeng W., Jin L., Zhang F., Zhang C., Liang W. (2018). Naringenin as a potential immunomodulator in therapeutics. Pharmacol. Res..

[61] Miles E.A., Calder P.C. (2021). Effects of Citrus Fruit Juices and Their Bioactive Components on Inflammation and Immunity: A Narrative Review. Front. Immunol..

[62] Shi Y., Zhou J., Jiang B., Miao M. (2017). Resveratrol and inflammatory bowel disease. Ann. N. Y. Acad. Sci..

[63] Rashidi R., Rezaee R., Shakeri A., Hayes A.W., Karimi G. (2022). A review of the protective effects of chlorogenic acid against different chemicals. J. Food Biochem..

[64] Kim S.R., Jung Y.R., An H.J., Kim D.H., Jang E.J., Choi Y.J., Moon K.M., Park M.H., Park C.H., Chung K.W. (2013). Anti-wrinkle and anti-inflammatory effects of active garlic components and the inhibition of MMPs via NF-κB signaling. PLoS ONE.

[65] Duan L., Cheng S., Li L., Liu Y., Wang D., Liu G. (2021). Natural Anti-Inflammatory Compounds as Drug Candidates for Inflammatory Bowel Disease. Front. Pharmacol..

[66] Zhang H., Deng A., Zhang Z., Yu Z., Liu Y., Peng S., Wu L., Qin H., Wang W. (2016). The protective effect of epicatechin on experimental ulcerative colitis in mice is mediated by increasing antioxidation and by the inhibition of NF-κB pathway. Pharmacol. Rep..

[67] Fitzpatrick L.R., Wang J., Le T. (2001). Caffeic acid phenethyl ester, an inhibitor of nuclear factor-kappaB, attenuates bacterial peptidoglycan polysaccharide induced colitis in rats. J. Pharmacol. Exp. Ther..

[68] Liu S.H., Lu T.H., Su C.C., Lay I.S., Lin H.Y., Fang K.M., Ho T.J., Chen K.L., Su Y.C., Chiang W.C. (2014). Lotus leaf (Nelumbo nucifera) and its active constituents prevent inflammatory responses in macrophages via JNK/NF-κB signaling pathway. Am. J. Chin. Med..

[69] Sunil M.A., Sunitha V.S., Santhakumaran P., Mohan M.C., Jose M.S., Radhakrishnan E.K., Mathew J. (2021). Protective effect of (+)-catechin against lipopolysaccharide-induced inflammatory response in RAW 264.7 cells through downregulation of NF-κB and p38 MAPK. Inflammopharmacology.

[70] Gonzales A.M., Orlando R.A. (2008). Curcumin and resveratrol inhibit nuclear factor-kappaB-mediated cytokine expression in adipocytes. Nutr. Metab. (Lond.).

[71] Lim R., Barker G., Wall C.A., Lappas M. (2013). Dietary phytophenols curcumin, naringenin and apigenin reduce infection-induced inflammatory and contractile pathways in human placenta, foetal membranes and myometrium. Mol. Hum. Reprod..

[72] Yang H.L., Chen S.C., Senthil Kumar K.J., Yu K.N., Lee Chao P.D., Tsai S.Y., Hou Y.C., Hseu Y.C. (2012). Antioxidant and anti-inflammatory potential of hesperetin metabolites obtained from hesperetin-administered rat serum: An ex vivo approach. J. Agric. Food Chem..

[73] Coates M.D., Mahoney C.R., Linden D.R., Sampson J.E., Chen J., Blaszyk H., Crowell M.D., Sharkey K.A., Gershon M.D., Mawe G.M. (2004). Molecular defects in mucosal serotonin content and decreased serotonin reuptake transporter in ulcerative colitis and irritable bowel syndrome. Gastroenterology.

[74] Minderhoud I.M., Oldenburg B., Schipper M.E.I., ter Linde J.J.M., Samsom M. (2007). Serotonin synthesis and uptake in symptomatic patients with Crohn’s disease in remission. Clin. Gastroenterol. Hepatol..

[75] Stoyanova I.I., Gulubova M.V. (2002). Mast cells and inflammatory mediators in chronic ulcerative colitis. Acta Histochem..

[76] Bearcroft C.P., Perrett D., Farthing M.J.G. (1998). Postprandial plasma 5-hydroxytryptamine in diarrhoea predominant irritable bowel syndrome: A pilot study. Gut.

[77] Spiller R.C., Jenkins D., Thornley J.P., Hebden J.M., Wright T., Skinner M., Neal K.R. (2000). Increased rectal mucosal enteroendocrine cells, T lymphocytes, and increased gut permeability following acute Campylobacter enteritis and in post-dysenteric irritable bowel syndrome. Gut.

[78] Chen M., Gao L., Chen P., Feng D., Jiang Y., Chang Y., Jin J., Chu F.F., Gao Q. (2016). Serotonin-Exacerbated DSS-Induced Colitis Is Associated with Increase in MMP-3 and MMP-9 Expression in the Mouse Colon. Mediat. Inflamm..

[79] Brunetti L., Orlando G., Ferrante C., Recinella L., Leone S., Chiavaroli A., Di Nisio C., Shohreh R., Manippa F., Ricciuti A. (2013). Orexigenic effects of omentin-1 related to decreased CART and CRH gene expression and increased norepinephrine synthesis and release in the hypothalamus. Peptides.

[80] Brunetti L., Orlando G., Ferrante C., Recinella L., Leone S., Chiavaroli A., Di Nisio C., Shohreh R., Manippa F., Ricciuti A. (2014). Peripheral chemerin administration modulates hypothalamic control of feeding. Peptides.

[81] Lee J., Chang C., Liu I., Chi T., Yu H., Cheng J. (2001). Changes in endogenous monoamines in aged rats. Clin. Exp. Pharmacol. Physiol..

[82] Menghini L., Ferrante C., Leporini L., Recinella L., Chiavaroli A., Leone S., Pintore G., Vacca M., Orlando G., Brunetti L. (2016). An Hydroalcoholic Chamomile Extract Modulates Inflammatory and Immune Response in HT29 Cells and Isolated Rat Colon. Phytother. Res..

[83] Ferrante C., Recinella L., Ronci M., Orlando G., Di Simone S., Brunetti L., Chiavaroli A., Leone S., Politi M., Tirillini B. (2019). Protective effects induced by alcoholic Phlomis fruticosa and Phlomis herba-venti extracts in isolated rat colon: Focus on antioxidant, anti-inflammatory, and antimicrobial activities in vitro. Phytother. Res..

[84] Batshaw M.L., Hyman S.L., Coyle J.T., Robinson M.B., Qureshi I.A., Mellits E.D., Quaskey S. (1988). Effect of sodium benzoate and sodium phenylacetate on brain serotonin turnover in the ornithine transcarbamylase-deficient sparse-fur mouse. Pediatr. Res..

[85] Li Y., Yao J., Han C., Yang J., Chaudhry M.T., Wang S., Liu H., Yin Y. (2016). Quercetin, Inflammation and Immunity. Nutrients.

[86] Tian T., Wang Z., Zhang J. (2017). Pathomechanisms of Oxidative Stress in Inflammatory Bowel Disease and Potential Antioxidant Therapies. Oxid. Med. Cell. Longev..

[87] Bourgonje A.R., Feelisch M., Faber K.N., Pasch A., Dijkstra G., van Goor H. (2020). Oxidative Stress and Redox-Modulating Therapeutics in Inflammatory Bowel Disease. Trends Mol. Med..

[88] Brückner M., Westphal S., Domschke W., Kucharzik T., Lügering A. (2012). Green tea polyphenol epigallocatechin-3-gallate shows therapeutic antioxidative effects in a murine model of colitis. J. Crohns Colitis.

[89] Praticò D., Lee V.M.Y., Trojanoswki J.Q., Rokach J., FitzGerald G.A. (1998). Increased F2-isoprostanes in Alzheimer’s disease: Evidence for enhanced lipid peroxidation in vivo. FASEB J..

[90] Petropoulos S., Fernandes Â., Barros L., Ciric A., Sokovic M., Ferreira I.C. (2018). Antimicrobial and antioxidant properties of various Greek garlic genotypes. Food Chem..

