# Supplementary material for: Anti-Inflammatory and Antioxidant Effects Induced by Allium sativum L. Extracts on an Ex Vivo Experimental Model of Ulcerative Colitis
Source: Foods. 2022 Nov 9;11(22):3559. doi: 10.3390/foods11223559 (PMC9689397; doi:10.3390/foods11223559)
Supplement: Supplementary file 1 [file foods-11-03559-s001.zip › foods-1929505-supplementary.pdf]

Anti-Inflammatory and Antioxidant Effects Induced by *Allium sativum* L. Extracts on an Ex Vivo Experimental Model of Ulcerative Colitis

Lucia Recinella [1], Era Gorica [2], Annalisa Chiavaroli [1,*], Caterina Fraschetti [3], Antonello Filippi [3], Stefania Cesa [3], Francesco Cairone [3], Alma Martelli [2,4,5], Vincenzo Calderone [2,4,5], Serena Veschi [1], Paola Lanuti [6], Alessandro Cama [1], Giustino Orlando [1], Claudio Ferrante [1], Luigi Menghini [1], Simonetta Cristina Di Simone [1], Alessandra Acquaviva [1,7], Maria Loreta Libero [1], Nilofar Nilofar [1], Luigi Brunetti [1] and Sheila Leone [1,*]

1 Department of Pharmacy, G. d'Annunzio University of Chieti-Pescara, 66013 Chieti, Italy
2 Department of Pharmacy, University of Pisa, 56126 Pisa, Italy
3 Department of Drug Chemistry and Technology, Sapienza University of Rome, 00185 Rome, Italy
4 Interdepartmental Research Center "Nutrafood: Nutraceutica e Alimentazione per la Salute", University of Pisa, 56126 Pisa, Italy
5 Interdepartmental Research Center "Biology and Pathology of Ageing", University of Pisa, 56126 Pisa, Italy
6 Department of Medicine and Aging Science, Centre on Aging Sciences and Translational Medicine (Ce.S.I-MeT), University "G. d'Annunzio" of Chieti-Pescara, 66100 Chieti, Italy
7 Veridia Italia Srl, Via Raiale 285, 65100 Pescara, Italy
* Correspondence: annalisa.chiavaroli@unich.it (A.C.); sheila.leone@unich.it (S.L.); Tel.: +39-0871-3554658 (A.C.); +39-0871-3554774 (S.L.)

High Performance Liquid Chromatography (HPLC)-Diode Array (DAD)-Mass Spectrometry (MS) Analysis

The extracts were analyzed for phenol quantitative determination using a reversed-phase HPLC-DAD-MS in gradient elution mode. The separation was conducted within 60 min of the chromatographic run, starting from the following separation conditions: 97% water with 0.1% formic acid, 3% methanol with 0.1% formic acid, as previously reported. The separation was performed on an Infinity lab Poroshell 120-SB reverse phase column (C18, 150 × 4.6 mm i.d., 2.7 μm) (Agilent, Santa Clara, CA, USA). Column temperature was set at 30 °C. Quantification was done through 7-point calibration curves, with linearity coefficients (R2) > 0.999, in the concentration range 2–140 μg/mL. All standards were purchased from Sigma Aldrich (Milan, Italy), and have a purity ≥ 95 %. The limits of detection were lower than 1 μg/mL for all assayed analytes. The area under the curve from HPLC chromatograms was used to quantify the analyte concentrations in the extract. The extracts were also qualitatively analyzed using an expression compact mass spectrometer (Advion, Ithaca, NY, USA) in negative and positive ion mode (m/z scan mode: 100–1200). MS signal identification was realized through comparison with a standard solution and MS spectra present in the MassBankEurope database. The details of the analysis are reported in supplementary tables.

Table S1. MS analysis conditions

Ion Source Parameters (APCI Negative):	**Ion Source Parameters (APCI Positive):**
Capillary Temperature = 250 °C	**Capillary Temperature = 250 °C**
Capillary Voltage = 120 V	**Capillary Voltage = 140 V**
Source Voltage Offset = 20 V	**Source Voltage Offset = 30 V**
Source Voltage Span = 30 V	**Source Voltage Span = 10 V**
APCI Source:	**APCI Source:**
Source Gas Temperature = 250 °C	**Source Gas Temperature = 250 °C**
Transfer Line Temperature = 100 °C	**Transfer Line Temperature = 100 °C**
APCI Corona Discharge = 3 μA	**APCI Corona Discharge = 3 μA**
Tune Parameters Voltages:	**Tune Parameters Voltages:**
Extraction Electrode = 9	**Extraction Electrode = 9**
Hexapole Bias = 8	**Hexapole Bias = 8**
Hexapole RF Offset = 5	**Hexapole RF Offset = 5**
Hexapole RF Span = 150	**Hexapole RF Span = 150**
Ion Energy Offset = -1.2	**Ion Energy Offset = -1.2**
Ion Energy Span = -0.4	**Ion Energy Span = -0.4**
Resolution Offset= -0.01	**Resolution Offset= -0.02**
Resolution Span = -1.42	**Resolution Span = -1.7**
Detector gain = 1300	**Detector gain = 1200**

Table S2. Retention times, wavelengths of quantification, mass to charge (*m*/*z*) ratios, and molecular weight of the investigated phenolic compounds.

**Table S2**					
**Standard**	Retention Time (min)	Wavelength (nm)	*m*/*z* ratio (negative ione mode)	*m*/*z* ratio (positive ione mode)	mw (g/mol)
Gallic acid	8.947	254	169.15	171.15	170.15
3-Hydroxytyrosol	11.923	254	153.16	155.16	154.16
Caftaric acid	12.823	254	311.23	313.23	311.23
Catechin	15.383	254	289.27	291.27	290.27
4-Hydroxybenzoic acid	16.703	254	137.12	139.12	138.12
Loganic acid	17.167	254	375.36	377.36	376.36

Chlorogenic acid	17.617	254	353.31	355.31	354.31
Vanillic acid	19.21	254	167.15	169.15	168.15
Caffeic acid	19.647	254	179.16	181.16	170.16
Epicatechin	20.083	254	289.27	290.27	290.27
Syringaldehyde	22.443	254	181.17	183.17	182.17
p-Coumaric acid	23.7	254	163.16	165.16	164.16
t-Ferulic acid	24.73	254	193.18	195.18	194.18
Benzoic acid	27.09	254	121.12	123.12	122.12
Rutin	28.303	254	609.52	611.52	610.52
Resveratrol	28.727	254	227.24	229.24	228.24
t-Cinnamic acid	35.983	254	147.15	149.15	148.15
Quercetin	38.137	254	301.23	303.23	302.23
Naringenin	38.96	254	271.25	273.25	272.25
Hesperetin	41.04	254	301.28	303.28	302.28
Kaempferol	42.567	254	285.24	287.24	286.24
Carvacrol	44.303	254	149.22	151.22	150.22
Thymol	44.42	254	149.22	151.22	150.22
Flavone	45.153	254	221.34	223.34	222.34
3-Hydroxyflavone	45.48	254	237.24	239.24	238.24
Emodin	47.057	254	269.24	271.24	270.24

Table S3. Gradient elution conditions of the HPLC-DAD analyses for investigated polyphenolic compounds in garlic hydroalcoholic and water extracts (GHE and GWE).

**Table S3.**		
**Time (min)**	**Water +0.1% Formic Acid**	**Methanol +0.1% Formic Acid**
1	97%	3%
5	77	23
12	73	27
18	57	43
25	52	48
32	50	50
34	50	50
37	35	65
40	5	95
47	5	95
48	97	3
flow: 0.6 mL/min		

HPLC-DAD Analysis

The aqueous extract (GWE) was weighed, dissolved in water and filtered before injection into a HPLC Perkin Elmer (Milan, Italy) apparatus (Series 200 LC pump, Series 200 DAD and Series 200 autosampler). Data acquisition and processing were carried out with a Perkin-Elmer Totalchrom software. A Luna RP18 column (150 × 4.6 mm, i.d. 3 μm) and a mobile phase, consisting of acetonitrile (A) and acidic water solution at 5% of formic acid (B) in gradient, were used, from 100% aqueous phase to 85% in 15 min, 85 to 55% in 30′ and 55 to 40% in 20′, at a flow rate of 0.8 mL/min. The detection wavelengths were set at 254 nm for the detection of sulfur and phenolic compounds. The injection volume for each extract was 10 μL. Alliin, identified at 254 nm in each sample, was quantified by an external standard matched calibration method on the basis of the area ratios respect to the pure chemical standard ($y = 8.96x + 41.84$; $R^2 = 0.9974$).

Colorimetric Analysis

CIEL*a*b parameters (L*, a*, b*) were determined directly on the garlic powder (GP) sample and the aqueous extract (GWE) using a colorimetric X-Rite MetaVueTM® (Prato, Italy) equipped with full-spectrum LED illuminant and an observer angle of 45°/0° imaging spectrophotometer [15]. Cylindrical coordinates C^*_{ab} and h_{ab} were calculated from a* and b* by Eqs (1) and (2):

(1) $C^*_{ab} = (a^{*2} + b^{*2})^{1/2}$;

(2) $h_{ab} = \tan^{-1} (b^*/a^*)$;

The garlic powder sample and the aqueous extract analysis was performed at the time of delivery (t°) and after 12 months (t^{12m}) of storage in the darkness at room temperature (25 ± 2 °C).

RNA extraction

Total RNA was extracted from the colon specimens using TRI Reagent (Sigma–Aldrich, St. Louis, MO), according to the manufacturer's instructions. Briefly, each colon sample was homogenized in 1 ml of TRI Reagent. The homogenate was centrifuged at 12,000 × g for 10 min at 4 ◦C to remove the insoluble material. The supernatant, containing RNA and proteins, was added to 0.2 ml chloroform, then shaken vigorously and incubated on ice for 15 min. The next centrifugation at 12,000 × g for 15 min at 4 ◦C separated the mixture into three phases: a red organic phase, containing proteins, an interphase, containing DNA, and a colorless upper aqueous phase, containing RNA. The aqueous phase was removed and RNA was precipitated with 0.5 ml of isopropanol, stored for 30 min at −20 ◦C and pelleted by centrifugation at 12,000 × g for 10 min at 4 ◦C. The RNA pellet was washed with 1 ml of 75% ethanol, air dried and finally resuspended in RNase-free water. Contaminating DNA was removed using 2 units of RNase-free DNase-1 (DNA-free kit, Ambion, Austin, TX), according to the manufacturer's protocol. RNA purity and concentration were determined by spectrophotometry (BioPhotometer Eppendorf, Hamburg, Germany). In particular, RNA concentration was determined by spectrophotometer reading at 260 nm and its purity was assessed by the ratio at 260 and 280 nm readings. In order to evaluate the quality of extracted RNA, the samples were tested by electrophoresis through agarose gels and visualized by staining with ethidium bromide, under UV light. 2.4.

Reverse transcription and real-time reverse transcription polymerase chain reaction (real-time RT PCR)

High capacity cDNA Reverse Transcription Kit (ThermoFischer Scientific, Waltman, Massachusetts, USA) was used to reverse transcribe RNA extracted from each colon specimen. In order to reverse transcribe 1 µg of RNA in a reaction volume of 20 µl, the reaction master mix was prepared according to the manufacturer's protocol. Reactions were incubated in a 2720 Thermal Cycler (ThermoFischer Scientific, Waltman, Massachusetts, USA) initially at 25 ◦C for 10 min, then at 37 ◦C for 120 min and finally at 85 ◦C for 5 s. Gene expression was determined by quantitative real-time PCR using TaqMan probe-based chemistry (ThermoFischer Scientific, Waltman, Massachusetts, USA). Reactions were performed in MicroAmp Fast Optic 96-well Reaction Plates (ThermoFischer Scientific, Waltman, Massachusetts, USA) on an ABI PRISM 7900 HT Fast Real-Time PCR System (ThermoFischer Scientific, Waltman, Massachusetts, USA), in "fast" operational mode. TaqMan probes and PCR primers were obtained from ThermoFisher Scientific [TaqMan Gene Expression Assays (20x)—Assays-on-Demand: Mm00478374_m1 for cyclooxygenase (COX)-2 gene, Mm00443258_m1 for tumor necrosis factor (TNF)-α gene, Mm00476361_m1 for nuclear factor kappa light chain enhancer of activated B cells (NF-kB) gene, Mm00446190_m1 for interleukin (IL)-6 gene, Mm00477784_m1 for nuclear factor erythroid 2–related factor 2 (Nrf2) gene, Mm00607939_s1 for β-actin gene)]. β-actin was used as the housekeeping gene. Each amplification reaction was performed with 10 µl of TaqMan Fast Universal PCR Master Mix (2×), No AmpErase UNG (ThermoFischer Scientific, Waltman, Massachusetts, USA), 1 µl of primer probe mixture, 1 µl of cDNA and 8 µl of nuclease-free water, according to the manufacturer's protocol. Real-time PCR was carried out in triplicate for each cDNA sample in relation to each of the investigated genes. No-template control, one for each Assay-on-Demand, was used to check for contamination. A reverse transcriptase minus control was included for β-actin gene Assay-on-Demand. The thermal cycling conditions were: 95 ◦C for 20 s, followed by 40 cycles of amplification at 95 ◦C for 1 s and 60 ◦C for 20 s. Sequence Detection System (SDS) software – version 2.3 – (ThermoFischer Scientific, Waltman, Massachusetts, USA) elaborated gene expression data. The comparative $2^{-\Delta\Delta Ct}$ method was used to quantify the relative abundance of mRNA and then determine the relative changes in individual gene expression (relative quantification, RQ). This method uses a calibrator sample to enable a comparison of gene expression levels in different samples. The values obtained indicate the changes in gene expression in the sample of interest by comparison with the calibrator sample, after normalization to the housekeeping gene.
